# Congenital Hyperinsulinism and Novel *KDM6A* Duplications -Resolving Pathogenicity With Genome and Epigenetic Analyses

**DOI:** 10.1210/clinem/dgae524

**Published:** 2024-07-30

**Authors:** Jonna M E Männistö, Jasmin J Hopkins, Thomas I Hewat, Fatima Nasser, Joseph Burrage, Antonia Dastamani, Alice Mirante, Nuala Murphy, Jessica Rzasa, Jennifer Kerkhof, Raissa Relator, Matthew B Johnson, Thomas W Laver, Luke Weymouth, Jayne A L Houghton, Matthew N Wakeling, Bekim Sadikovic, Emma L Dempster, Sarah E Flanagan

**Affiliations:** Department of Clinical and Biomedical Science, University of Exeter Medical School, Exeter EX2 5DW, UK; Kuopio Pediatric Research Unit, Faculty of Health Sciences, School of Medicine, University of Eastern Finland, 70211 Kuopio, Finland; Department of Clinical and Biomedical Science, University of Exeter Medical School, Exeter EX2 5DW, UK; Department of Clinical and Biomedical Science, University of Exeter Medical School, Exeter EX2 5DW, UK; Department of Clinical and Biomedical Science, University of Exeter Medical School, Exeter EX2 5DW, UK; Department of Clinical and Biomedical Science, University of Exeter Medical School, Exeter EX2 5DW, UK; Endocrinology Department, Great Ormond Street Hospital for Children, London WC1N 3JH, UK; Pediatric Endocrinology, Hospital Pediátrico de Coimbra, ULS de Coimbra, 3000-602 Coimbra, Portugal; Department of Paediatric Endocrinology, CHI Temple St, Dublin, D01 XD99, Ireland; Verspeeten Clinical Genome Centre, London Health Sciences Centre, London, ON N6C 2R6, Canada; Verspeeten Clinical Genome Centre, London Health Sciences Centre, London, ON N6C 2R6, Canada; Verspeeten Clinical Genome Centre, London Health Sciences Centre, London, ON N6C 2R6, Canada; Department of Clinical and Biomedical Science, University of Exeter Medical School, Exeter EX2 5DW, UK; Department of Clinical and Biomedical Science, University of Exeter Medical School, Exeter EX2 5DW, UK; Department of Clinical and Biomedical Science, University of Exeter Medical School, Exeter EX2 5DW, UK; Exeter Genomics Laboratory, Royal Devon University Healthcare NHS Foundation Trust, Exeter EX2 5DW, UK; Department of Clinical and Biomedical Science, University of Exeter Medical School, Exeter EX2 5DW, UK; Verspeeten Clinical Genome Centre, London Health Sciences Centre, London, ON N6C 2R6, Canada; Department of Pathology and Laboratory Medicine, Western University, London, ON N6A 5C1, Canada; Department of Clinical and Biomedical Science, University of Exeter Medical School, Exeter EX2 5DW, UK; Department of Clinical and Biomedical Science, University of Exeter Medical School, Exeter EX2 5DW, UK

**Keywords:** *KDM6A*, Kabuki syndrome, congenital hyperinsulinism, DNA methylation, episignature, whole-genome sequencing

## Abstract

**Context:**

Hyperinsulinemic hypoglycemia (HI) can be the presenting feature of Kabuki syndrome (KS), which is caused by loss-of-function variants in *KMT2D* or *KDM6A.* As these genes play a critical role in maintaining methylation status in chromatin, individuals with pathogenic variants have a disease-specific epigenomic profile—an episignature.

**Objective:**

We evaluated the pathogenicity of 3 novel partial *KDM6A* duplications identified in 3 individuals presenting with neonatal-onset HI without typical features of KS at the time of genetic testing.

**Methods:**

Three different partial *KDM6A* duplications were identified by routine targeted next-generation sequencing for HI and initially classified as variants of uncertain significance (VUS) as their location, and hence their impact on the gene, was not known. Whole-genome sequencing (WGS) was undertaken to map the breakpoints of the duplications with DNA methylation profiling performed in 2 individuals to investigate the presence of a KS-specific episignature.

**Results:**

WGS confirmed the duplication in proband 1 as pathogenic as it caused a frameshift in the normal copy of the gene leading to a premature termination codon. The duplications identified in probands 2 and 3 did not alter the reading frame, and therefore their significance remained uncertain after WGS. Subsequent DNA methylation profiling identified a KS-specific episignature in proband 2 but not in proband 3.

**Conclusion:**

Our findings confirm a role for *KDM6A* partial gene duplications in the etiology of KS and highlight the importance of performing in-depth molecular genetic analysis to properly assess the clinical significance of VUS' in the *KDM6A* gene.

Kabuki syndrome (KS) is a developmental disorder originally characterized by typical facial features, mild to moderate intellectual disability, minor specific skeletal and dermatoglyphic anomalies, and postnatal growth deficiency (1). The overlap in the KS phenotype with other monogenic developmental disorders together with the observation that many characteristic features are nonspecific and only become apparent later in childhood mean that a clinical diagnosis of KS can be challenging, especially in early infancy (2).

Pathogenic loss-of-function variants in *KMT2D* (autosomal dominant KS type 1, OMIM #147920) and *KDM6A* (X-linked dominant KS type 2, OMIM #300867) account for >80% and 6% to 10% of clinically diagnosed KS cases, respectively (3-5). Both genes encode enzymes that modify histones in the chromatin by demethylation/methylation. These changes in chromatin status serve to regulate the transcription of genes at a specific genomic location. Consequently, individuals with pathogenic variants in *KDM6A* or *KMT2D* have alterations to DNA methylation at more than 20 genomic regions, along with >1500 CpG sites across the genome with the most differentially methylated regions including *Hox* genes and *MYOF1* (6). The resulting pattern of disease-associated alterations in DNA methylation is referred to as an “episignature” and is considered an effective biomarker for a growing number of Mendelian disorders (6, 7).

High-throughput sequencing analysis has allowed for more rapid and accurate genetic diagnosis of individuals with KS. This has also served to expand the phenotypic spectrum of the condition, which is now recognized to manifest with a broad range of congenital anomalies and functional abnormalities, including endocrine dysfunction (2, 3).

A common endocrine condition observed in individuals with KS is hyperinsulinemic hypoglycemia (HI). This is more frequently associated with *KDM6A*-KS than *KMT2D*-KS (∼22% vs ∼4%, respectively) (8). As HI is often diagnosed very close to birth, it can be the presenting feature of KS (9), and consequently, many diagnostic laboratories include *KDM6A* and *KMT2D* on their targeted gene panels for HI testing (10).

*KMT2D* and *KDM6A* are highly polymorphic genes, with large numbers of pathogenic stop gain, frameshift, splice site variants, missense changes, and gross deletions described (3, 11, 12). Large intragenic duplications have also been reported in a few individuals with KS (13). For *KDM6A* this includes a single report of a tandem duplication of exon 3. In this case, whole-genome sequencing (WGS) confirmed pathogenicity by showing that the duplication disrupted the reading frame of the normal copy of the gene resulting in a loss-of-function (14).

For many laboratories, assessing the pathogenicity of novel *KMT2D* and *KDM6A* variants that do not clearly result in a loss-of-function (eg, missense changes, in-frame deletions/duplications, and large duplications) can be challenging, especially when the individual is young and may not have developed features of KS (15). The discovery of a disease-specific methylation profile or episignature for KS is, however, revolutionizing the ability to assess the pathogenicity of novel genetic variants within the diagnostic setting. By analyzing the methylation status of CpG positions across the genome and comparing this profile to KS and unaffected control cohorts, it is now possible to accurately predict whether an individual has KS due to a disruption of the *KDM6A* or *KMT2D* genes. The predictions can then be used in combination with additional genetic and clinical data to help discriminate whether a variant is likely to be pathogenic or not (6, 16, 17).

In this study, we identified 3 large partial duplications of *KDM6A* in 3 individuals referred for routine genetic testing for HI without a clinical suspicion of KS. The duplications were initially considered variants of uncertain significance but were subsequently reclassified following WGS and/or epigenomic profiling.

## Methods

### Participants

The 3 individuals were referred to the Exeter Genomics Laboratory for routine genetic testing for HI. Clinical data were collected from standardized referral forms with follow-up information obtained by case-note review from the treating clinicians. Informed consent was obtained from each of the parents with the study approved by the North Wales Research Ethics Committee (517/WA/0327).

### Sequencing Analysis

Initial testing involved targeted next-generation sequencing (tNGS) of the coding regions of 16 known HI genes (*ABCC8*, *KCNJ11*, *GLUD1*, *HNF4A*, *GCK*, *HADH*, *INSR*, *SLC16A1*, *TRMT10A*, *HNF1A*, *CACNA1D*, *GPC3*, *KDM6A*, *KMT2D*, *MAFA*, and *PMM2)* using DNA extracted from peripheral blood leukocytes following previously reported methods (18). This analysis also allows for calling of on-target copy number variations (CNVs) using read depth analysis.

WGS was undertaken using Illumina HiSeq, Illumina TruSeq, or BGISeq-500 technology to confirm duplication breakpoints in all 3 probands. Sequence data were aligned with BWA MEM 0.7.15 and processed using a pipeline based on the GATK best practices (19) (Picard version 2.7.1, GATK version 3.7). Variants were annotated using Alamut batch standalone v1.11 software (SOPHiA genetics, Lausanne, Switzerland). All genetic data were annotated using the Genome Reference Consortium Human Build 37 (accession number GCF_000001405.13).

### Whole-Genome Methylation Profiling

DNA methylation profiles of leukocyte DNA from 2 patients were generated using the Illumina EPIC DNA methylation array. Analysis was conducted using the clinically validated EpiSign assay, following previously established methods (6, 17, 20, 21). Methylated and unmethylated signal intensities generated from the EPIC array were imported into R 4.2.1 for normalization, background correction, and filtering. Beta values were then calculated as a measure of methylation level, ranging from 0 (no methylation) to 1 (complete methylation), and processed through the established support vector machine classification algorithm for EpiSign disorders. The classifier utilized the EpiSign Knowledge Database, which consists of more than 10 000 methylation profiles from reference disorder-specific and unaffected control cohorts, to generate disorder-specific methylation variant pathogenicity (MVP) scores. These MVP scores are a measure of prediction confidence for each disorder and range from 0 (discordant) to 1 (highly concordant). A positive classification typically generates MVP scores greater than 0.5. The final matched EpiSign result is generated using these scores, along with the assessment of hierarchical clustering and multidimensional scaling (22).

### Family Member Testing

Each proband´s CNV was confirmed de novo by testing leukocyte DNA from the unaffected biological parents using WGS (proband 1), droplet digital PCR (Bio-Rad QX200 system, with EvaGreen and primers targeted against multiple exons within *KDM6A*) (proband 2), or multiplex ligation-dependent probe amplification (SALSA MLPA Probe mix P445-A3 *KDM6A*) used according to the manufacturer's instructions (MRC-Holland, Amsterdam, the Netherlands) (proband 3). Methodological details are available on request.

### Variant Interpretation

The novel duplications were assessed according to the Association for Clinical Genomic Science best practice guidelines for variant classification in rare disease (23). The single nucleotide variant guidelines were used for interpreting the duplications with both breakpoints within the gene (24) (probands 1 and 2). The guidelines for interpretation of CNVs by the American College of Medical Genetics and Genomics and Clinical Genome Resource were used for interpreting the duplication without both breakpoints within the gene (25) (proband 3).

## Results

**Figure 1.**
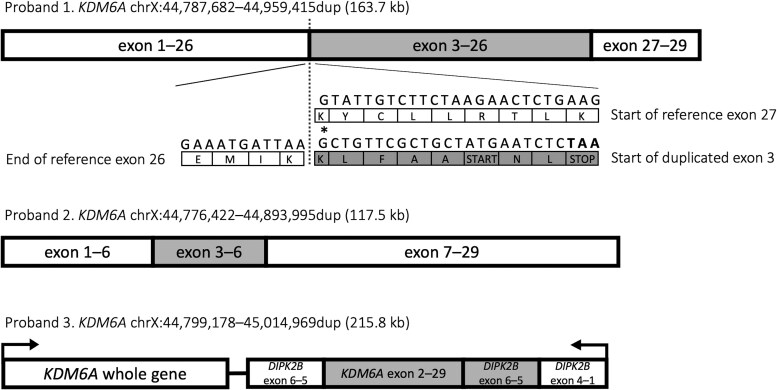
Duplications of the *KDM6A* gene identified in 3 individuals with hyperinsulinemic hypoglycemia by targeted next-generation sequencing with the breakpoints confirmed by genome sequencing. Variants are listed according to NM_021140.3, Genome Reference Consortium Human Build 37. A tandem duplication of exons 3-26 within the *KDM6A* gene causes a frameshift and results in a premature stop codon in the second copy of exon 3 (proband 1). An in-frame tandem duplication of exons 3-6 within the *KDM6A* gene (proband 2). A tandem duplication of exons 2-29 of *KDM6A* and exons 5-6 of the adjacent *DIPK2B* gene, located next to a complete copy of *KDM6A* within the *DIPK2B* gene (proband 3). Shaded grey indicates the duplications. Single letters within boxes indicate abbreviations of amino acids. Asterisk indicates the position of an introduced frameshift.Abbreviation: STOP, premature stop codon.

We identified 3 different large partial gene duplications in the *KDM6A* gene in 3 unrelated individuals using tNGS (Table 1, Fig. 1). As this method could not establish the genomic location of the duplicated sequence, the impact of the duplications on the normal copy of *KDM6A* could not be determined. The phenotype of the patients was also not specific for KS, and consequently the clinical significance of the 3 duplications was not known.

**Table 1.** Clinical features and genomic data from the 3 individuals with partial duplications of the *KDM6A* gene along with variant interpretation scores

	Proband 1	Proband 2	Proband 3
Genomic data (all coordinates related to GRCh37)			
tNGS Result and variant interpretation	*KDM6A* duplication of exons 3-26	*KDM6A* duplication of exons 3-6	*KDM6A* duplication of exons 2-29
	Uncertain	Uncertain	Uncertain
WGS Result and updated variant interpretation	163.7 kb tandem duplication mapping within *KDM6A* (ChrX:44,787,682–44,959,415dup) Resulting in a frameshift and premature stop codon	117.5 kb tandem duplication mapping within *KDM6A* (ChrX:44,776,422–44,893,995dup) Predicted to cause an in-frame duplication	215.8 kb tandem duplication mapping within *DIPK2B* (ChrX:44,799,178–45,014,969dup) Not predicted to disrupt the normal copy of *KDM6A*
	Pathogenic (PVS1_very strong PS2_Strong PM2_Supporting)	VUS (PS2_Strong PM2_Supporting)	VUS (4C: 0.15 points)
EPIC array analysis Result and updated variant interpretation	Not done	Consistent with KS	Inconsistent with KS
	NA	Likely pathogenic (PS2_Strong PM2_Supporting PP4_Supporting)	VUS (4D: −0.3points)
Clinical features			
Sex	Male	Female	Female
Birth weight, g (gestational age, weeks)	3760 (40)	3225 (39)	2600 (41)
Birth weight SDS	0.69	−0.23	−2.36
Age at last follow-up	4.5 years	3.7 years	7.5 years
Age at onset of hypoglycemia	1 day	1 day	3 weeks
Current treatment for hyperinsulinism	Diazoxide 5 mg/kg/d	None (diazoxide ∼5 mg/kg/d until aged 3.4 years)	None (diazoxide ∼4 mg/kg/d until aged 2 years)
Additional clinical features by the time of latest follow-up	Umbilical hernia, DD, ASD, learning difficulties, hypomobility, motor deficit, mild PVL, features consistent with KS	Mild birth asphyxia, congenital hip dislocation, mild global DD, postnatal growth delay, mild facial features	Congenital hypoplastic R-heart syndrome, birth asphyxia, global DD, autism

Duplications are reported according to NM_021140.3 with genomic coordinates listed according to GRCh37.

Variant classification using (23-25): PVS1_very strong: Duplication proven in tandem, reading frame disrupted, and nonsense-mediated decay predicted to occur. PS2_Strong: Confirmed de novo. PM2_Supporting: absent from population databases. PP4_Supporting: Patients phenotype is highly specific for the disease (episignature confirmed by methylation analysis). 4C: de novo, 4D: the reported phenotype (episignature confirmed by methylation analysis) is not consistent with the gene.

Abbreviations: ASD, autism spectrum disorder; DD, developmental delay; GA, gestational age; GRCh37, Genome Reference Consortium Human Build 37; KS, Kabuki syndrome; PVL, periventricular leukomalacia; NA, not applicable; SDS, SD score; tNGS, targeted next-generation sequencing; VUS, variant of unknown significance; WGS, whole genome sequencing.

### Proband 1

The male proband was born at 40 weeks gestation weighing 3760 g [0.69 SD scores (SDS)]. There was a history of diet-controlled gestational diabetes, fetal distress without birth asphyxia, and congenital umbilical hernia (Table 1). HI was diagnosed on the first day of life and showed a rapid response to diazoxide treatment. The patient was referred for routine screening of the known HI genes at the age of 2 weeks, which identified a hemizygous duplication of exons 3-26 of *KDM6A*.

At the age of 4.5 years, the HI was being treated successfully with 5 mg/kg/day of diazoxide. Developmental delay, autistic spectrum disorder with sensory problems, learning difficulties, hypomobility, and significant motor deficits were observed. Brain magnetic resonance scanning showed a possible mild periventricular leukomalacia inconsistent with hypoglycemic injury and not explaining the proband’s developmental presentations. Initially, no distinct facial dysmorphism was reported. Growth was within the average range at 6 years of age (height around −0.67 SDS). The results of genetic testing for Fragile X and Beckwith–Wiedemann syndromes and microarray were normal.

Given the development of these additional features, the significance of the *KDM6A* duplication was reconsidered. WGS was performed on samples from the child and both parents, which confirmed a de novo 163.7 kb duplication (ChrX:44,787,6824787682–44,959,4154959415dup). This duplication included exons 3-26 of *KDM6A,* which was inserted between exons 26 and 27 of the normal copy of the gene (Fig. 1). As the end of exon 26 shares a split codon with the start of exon 27, the duplication is predicted to introduce a frameshift at the beginning of the second copy of exon 3 leading to a premature stop codon at the eighth residue of exon 3. A full copy of the *KDM6A* protein is therefore not predicted to be produced as the mRNA would be targeted for nonsense-mediated decay. The duplication was subsequently upgraded to pathogenic (Table 1). At follow-up, the clinical features of the proband were confirmed by a clinical geneticist to be consistent with KS.

### Proband 2

This female was born at 39 weeks gestation weighing 3225 g (−0.23 SDS). She had mild birth asphyxia and congenital hip dislocation. On the first day of life, she presented with HI, which responded to diazoxide (4.7 mg/kg/d). At the age of 6 months, genetic testing for HI was undertaken, which identified a heterozygous duplication of exons 3-6 of *KDM6A*. Testing of parental samples by droplet digital PCR confirmed that the duplication had arisen de novo. No other clinical features were noted at that time.

WGS was performed, which confirmed a 117.5 kb in frame duplication (ChrX:44,776,422–44,893,995dup) inserted between exons 6 and 7 of the normal copy of *KDM6A* (Fig. 1). As the duplication was not predicted to impact on the reading frame of the normal copy of the gene and the phenotype was not specific for KS, the clinical significance of the duplication remained uncertain (Table 1).

**Figure 2.**
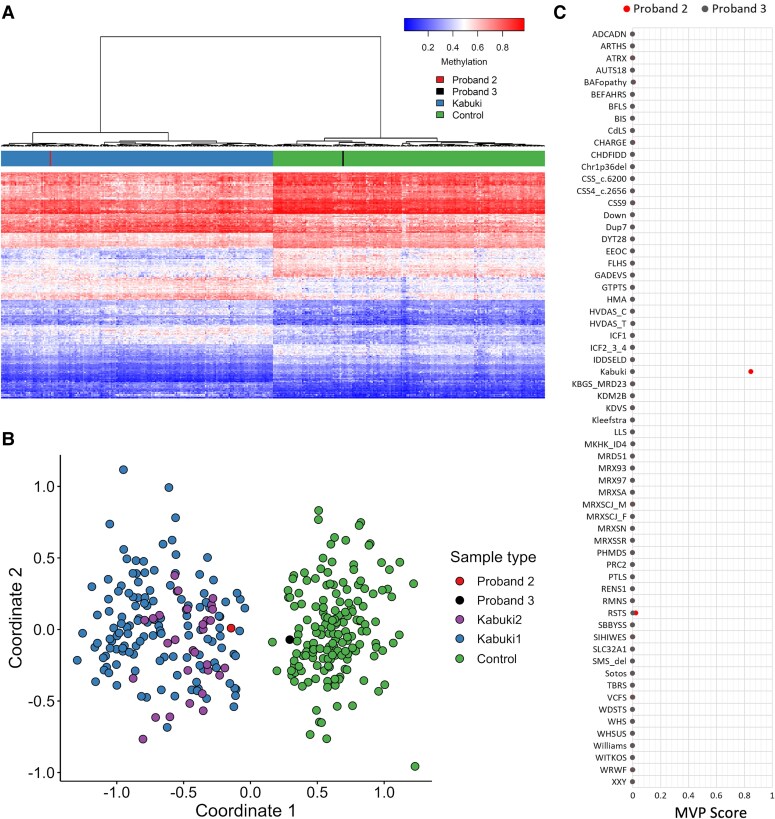
EpiSign (DNA methylation) analysis of peripheral blood from patient 2 and 3 with tandem duplications of *KDM6A*. (A) Hierarchical clustering. The plot shows clustering analysis with heatmap using probes specific to the DNA methylation of KS as compared to controls. Rows indicate probes and columns indicate samples. (B) Multidimensional scaling. The 2 dimensions represent the pairwise distance across the samples with episignatures of KMD6A-KS patients (purple), KMT2D-KS patients (blue), and controls (green). Together these results indicate that Proband 2 (red line or plot) has a DNA methylation profile similar to subjects with a confirmed KS episignature (blue or purple) and distinct from controls (green). Proband 3 (black) has a DNA methylation profile similar to controls (green). C) MVP score. A multiclass supervised classification system capable of discerning between multiple episignatures by generating a probability score for each episignature. The elevated score for Kabuki shows an episignature similar to the KS reference. MVP score >0.5 indicates positive classification. Abbreviations: KS, Kabuki syndrome; MVP, methylation variant pathogenicity.

EPIC array analysis was subsequently performed, which showed that the DNA methylation profile of the proband was concordant with KS patients as indicated by Euclidean clustering, multidimensional scaling, and an elevated MVP score (0.847) (Fig. 2). This finding of an episignature consistent with KS supported the duplication being disease-causing.

At 3 years 5 months of age, diazoxide was successfully discontinued. By that age, the patient had been observed to show mild global developmental delay and mild facial dysmorphism. Additionally, the patient had significant postnatal growth failure with height −3.9 SDS at the age of 3.6 years. The findings in brain magnetic resonance imaging were normal. In light of the new genetic and clinical data, the *KDM6A* variant was reclassified as likely pathogenic (Table 1).

### Proband 3

The female proband was born at 41 weeks gestation weighing 2600 g (−2.36 SDS) (Table 1). There was a prenatal diagnosis of right-sided hypoplastic heart and perinatal asphyxia possibly secondary to difficult extraction. Hypoplastic R-heart syndrome was confirmed after birth; this feature is not typical of KS. She had the first cardiac operation on day 10 and subsequently developed necrotizing enterocolitis followed by a septic episode. Hypoglycemic episodes were observed at that time and initially considered as sepsis-related. At the age of 6 weeks, biochemistry suggested HI. Diazoxide treatment was started (4 mg/kg/day), and the patient was referred for genetic testing of the known HI genes, which identified a duplication of exons 2-29 of *KDM6A,* which was confirmed as de novo.

WGS was performed and showed a 215.8 kb duplication (ChrX:44,799,178–45,014,969dup), which included exons 2-29 of *KDM6A* and exons 6 and 5 of the adjacent gene, *DIPK2B* (Fig. 1). The duplicated sequence mapped within the *DIPK2B* gene and not *KDM6A*. As the phenotype was not specific for KDM6A-KS, the significance of the duplication remained unknown (Table 1).

EPIC array analysis was then performed on the sample from the proband, which demonstrated that the methylation profile was similar to controls with an MVP score of 0 for the KS episignature. The CNV interpretation score subsequently reduced from 0.15 to −0.30, and the significance of the variant remained unknown (Table 1).

By the age of 2 years, the patient was diagnosed with global developmental delay with nonverbal speech delay and autism, and diazoxide treatment for the hyperinsulinism was stopped. At the age of 7.5 years, no syndromic features consistent with KS were noted by a clinical geneticist, and the patient had no growth delay (height −1.3 SDS).

## Discussion

Using tNGS, we identified partial duplications of the *KDM6A* gene in 3 probands presenting with neonatal-onset HI. These 3 variants were initially classified as being of uncertain significance as the phenotype was not highly specific for KS and the location of the duplications, and thus their effect on the normal copy of the gene, was not known. By performing WGS, we established the location of the duplications. This allowed us to upgrade the duplication identified in proband 1 to pathogenic using American College of Medical Genetics/Association for Clinical Genomic Science criteria (23, 24). The 2 remaining duplications did not disrupt the reading frame of the normal copy of *KDM6A,* and hence their significance remained uncertain after WGS.

By performing epigenomic profiling, we were able to show that proband 2 had an episignature for KS confirming that the in-frame, tandem duplication was likely to be disrupting the normal copy of the *KDM6A* gene. In contrast, the absence of an episignature for KS in proband 3 suggested that the duplication was not causative of their HI. In this individual, the duplication resided within the adjacent gene, *DIPK2B*. While it is possible that a disruption of *DIPK2B* may have contributed to some of the clinical features in the patient, current evidence suggests that the duplication is likely to be benign given that *DIPK2B* has not been associated with human monogenic disease and the gene is not constrained for loss-of-function variants (GnomAD v2.1.1, pLI score: 0) (26).

Our findings confirm a role for large duplications, which disrupt the normal copy of *KDM6A*, in the etiology of KS. We were able to find only a single case with a large tandem duplication in the *KDM6A* gene in the literature. In this individual, a duplication of exon 3 resulted in an insertion of 109 bp causing a shift in the reading frame and hence was predicted to result in a loss-of-protein function (14). Interestingly, this variant was not identified by exome sequencing or copy number analysis and was only called on WGS following the identification of a KS-specific episignature. Taken with the findings of our study, this emphasizes the importance of studying epigenomic profiles in individuals with variants of uncertain significance in the KS genes or those with normal genetic results of *KMT2D* and *KDM6A* but presenting with KS-like disease (16, 17).

Our results highlight the difficulties that exist in interpreting large CNVs, especially large duplications whose breakpoints can remain undetermined by routine diagnostic screening methods such as tNGS (27). In these cases, it is not possible to determine whether the duplication is affecting the normal copy of the gene and hence whether there will be an impact on protein function. While we were able to perform WGS and epigenomic analysis to assess the duplications, we recognized that for many laboratories it is not feasible to perform these in-depth molecular investigations when a variant of uncertain significance is found. Furthermore, to generate a disorder-specific methylation variant pathogenicity score for an individual requires access to disorder-specific and unaffected control cohorts. For this study, we were able to collaborate with EpiSign, which has access to more than 10 000 methylation profiles, including individuals with KDM6A-KS and KMT2D-KS, allowing accurate scores to be generated for our 2 patients.

This study further highlights the difficulty in interpreting the significance of variants identified in individuals who may be too young to have developed features of a condition. None of the probands reported here presented in a way that would have seen them diagnosed with KS in a clinical setting according to the international consensus diagnostic criteria (2). All 3 had HI that presented soon after birth. As HI can be the presenting feature of KS and intellectual disability and facial dysmorphism are often not prominent until later in childhood, the absence of a clinical diagnosis of KS could not preclude the *KDM6A* variants being disease-causing. Moreover, several features are often milder or more infrequent in KDM6A-KS compared to KMT2D-KS, especially in females, most likely due to differences in X-chromosome inactivation (2, 11, 28). Our data support the inclusion of genes such as *KMT2D* and *KDM6A* in routine genetic testing for HI given that an early diagnosis of a syndromic condition could have beneficial long-term health implications such as earlier interventions for other comorbid conditions or developmental support.

In conclusion, we have shown that large partial gene duplications of *KDM6A* are an important cause of KS that may require further characterization by methylation profiling and/or WGS to establish their clinical significance. Our results support the need to include genes such as *KDM6A* on testing panels for HI but highlight the difficulties in interpreting novel variants whose impact on gene function is not immediately apparent, especially when identified in individuals who may be too young to have developed all the features of KS.

## Data Availability

Restrictions apply to the availability of some or all data generated or analyzed during this study to preserve patient confidentiality or because they were used under license. The corresponding author will on request detail the restrictions and any conditions under which access to some data may be provided. The *KDM6A* variants reported in this study were uploaded to the Decipher database (https://www.deciphergenomics.org/). Sequenicing data can be used to identify individuals and are therefore available only through collaboration to experienced teams working on approved studies examining the mechanisms, cause, diagnosis, and treatment of diabetes and other beta cell disorders. Requests for collaboration will be considered by a steering committee following an application to the Genetic Beta Cell Research Bank (https://www.diabetesgenes.org/current-research/genetic-beta-cell-research-bank/). Contact by email should be directed to S. Flanagan (s.flanagan@exeter.ac.uk). We used the Genome Reference Consortium Human Build 37 to annotate genetic data (accession number GCF_000001405.13). Details of this assembly are provided at https://www.ncbi.nlm.nih.gov/assembly/GCF_000001405.13/.

## Abbreviations

CNV: copy number variant
HI: congenital hyperinsulinism
KS: Kabuki syndrome
MVP: methylation variant pathogenicity
SDS: SD score
tNGS: targeted next-generation sequencing
WGS: whole-genome sequencing

## References

[1] Niikawa N , Kuroki Y, Kajii T, et al Kabuki make-up (Niikawa-Kuroki) syndrome: a study of 62 patients. Am J Med Genet. 1988;31(3):565‐589. 3067577 10.1002/ajmg.1320310312

[2] Adam MP , Banka S, Bjornsson HT, et al Kabuki syndrome: international consensus diagnostic criteria. J Med Genet. 2019;56(2):89‐95. 30514738 10.1136/jmedgenet-2018-105625

[3] Barry KK , Tsaparlis M, Hoffman D, et al From genotype to phenotype-a review of Kabuki syndrome. Genes (Basel). 2022;13(10):1761. 36292647 10.3390/genes13101761 PMC9601850

[4] Ng SB , Bigham AW, Buckingham KJ, et al Exome sequencing identifies MLL2 mutations as a cause of Kabuki syndrome. Nat Genet. 2010;42(9):790‐793. 20711175 10.1038/ng.646 PMC2930028

[5] Lederer D , Grisart B, Digilio MC, et al Deletion of KDM6A, a histone demethylase interacting with MLL2, in three patients with Kabuki syndrome. Am J Hum Genet. 2012;90(1):119‐124. 22197486 10.1016/j.ajhg.2011.11.021 PMC3257878

[6] Aref-Eshghi E , Schenkel LC, Lin H, et al The defining DNA methylation signature of Kabuki syndrome enables functional assessment of genetic variants of unknown clinical significance. Epigenetics. 2017;12(11):923‐933. 28933623 10.1080/15592294.2017.1381807 PMC5788422

[7] Sobreira N , Brucato M, Zhang L, et al Patients with a Kabuki syndrome phenotype demonstrate DNA methylation abnormalities. Eur J Hum Genet. 2017;25(12):1335‐1344. 29255178 10.1038/s41431-017-0023-0 PMC5865196

[8] Hoermann H , El-Rifai O, Schebek M, et al Comparative meta-analysis of Kabuki syndrome with and without hyperinsulinaemic hypoglycaemia. Clin Endocrinol (Oxf). 2020;93(3):346‐354. 32533869 10.1111/cen.14267

[9] Yap KL , Johnson AEK, Fischer D, et al Congenital hyperinsulinism as the presenting feature of Kabuki syndrome: clinical and molecular characterization of 9 affected individuals. Genet Med. 2019;21(1):233‐242. 29907798 10.1038/s41436-018-0013-9 PMC7597849

[10] Hewat TI , Johnson MB, Flanagan SE. Congenital hyperinsulinism: current laboratory-based approaches to the genetic diagnosis of a heterogeneous disease. Front Endocrinol. 2022;13:873254. 10.3389/fendo.2022.873254 PMC9302115 35872984

[11] Faundes V , Goh S, Akilapa R, et al Clinical delineation, sex differences, and genotype-phenotype correlation in pathogenic KDM6A variants causing X-linked Kabuki syndrome type 2. Genet Med. 2021;23(7):1202‐1210. 33674768 10.1038/s41436-021-01119-8 PMC8257478

[12] Bögershausen N , Gatinois V, Riehmer V, et al Mutation update for Kabuki syndrome genes KMT2D and KDM6A and further delineation of X-linked Kabuki syndrome subtype 2. Hum Mutat. 2016;37(9):847‐864. 27302555 10.1002/humu.23026

[13] Banka S , Veeramachaneni R, Reardon W, et al How genetically heterogeneous is Kabuki syndrome?: MLL2 testing in 116 patients, review and analyses of mutation and phenotypic spectrum. Eur J Hum Genet. 2012;20(4):381‐388. 22126750 10.1038/ejhg.2011.220 PMC3306863

[14] Marwaha A , Costain G, Cytrynbaum C, et al The utility of DNA methylation signatures in directing genome sequencing workflow: Kabuki syndrome and CDK13-related disorder. Am J Med Genet A. 2022;188(5):1368‐1375. 35043535 10.1002/ajmg.a.62650 PMC9303780

[15] Laver TW , Wakeling MN, Hua JHY, et al Comprehensive screening shows that mutations in the known syndromic genes are rare in infants presenting with hyperinsulinaemic hypoglycaemia. Clin Endocrinol (Oxf). 2018;89(5):621‐627. 30238501 10.1111/cen.13841 PMC6283248

[16] Aref-Eshghi E , Kerkhof J, Pedro VP, et al Evaluation of DNA methylation episignatures for diagnosis and phenotype correlations in 42 Mendelian neurodevelopmental disorders. Am J Hum Genet. 2020;106(3):356‐370. 32109418 10.1016/j.ajhg.2020.01.019 PMC7058829

[17] Levy MA , McConkey H, Kerkhof J, et al Novel diagnostic DNA methylation episignatures expand and refine the epigenetic landscapes of Mendelian disorders. HGG Adv. 2022;3(1):100075. 35047860 10.1016/j.xhgg.2021.100075 PMC8756545

[18] Ellard S , Lango Allen H, De Franco E, et al Improved genetic testing for monogenic diabetes using targeted next-generation sequencing. Diabetologia. 2013;56(9):1958‐1963. 23771172 10.1007/s00125-013-2962-5 PMC3737433

[19] Van der Auwera GA , O'Connor BD. Genomics in the Cloud: Using Docker, GATK, and WDL in Terra. 1st ed. O’Reilly Media; 2020.

[20] Sadikovic B , Levy MA, Kerkhof J, et al Clinical epigenomics: genome-wide DNA methylation analysis for the diagnosis of Mendelian disorders. Genet Med. 2021;23(6):1065‐1074. 33547396 10.1038/s41436-020-01096-4 PMC8187150

[21] Aref-Eshghi E , Bend EG, Colaiacovo S, et al Diagnostic utility of genome-wide DNA methylation testing in genetically unsolved individuals with suspected hereditary conditions. Am J Hum Genet. 2019;104(4):685‐700. 30929737 10.1016/j.ajhg.2019.03.008 PMC6451739

[22] Kerkhof J , Rastin C, Levy MA, et al Diagnostic utility and reporting recommendations for clinical DNA methylation episignature testing in genetically undiagnosed rare diseases. Genet Med. 2024;26(5):101075. 38251460 10.1016/j.gim.2024.101075

[23] Durkie M , Cassidy EJ, Berry I, et al ACGS Best Practice Guidelines for Variant Classification in Rare Disease 2024. Accessed 22 February 2024. https://www.acgs.uk.com/quality/best-practice-guidelines/

[24] Richards S , Aziz N, Bale S, et al Standards and guidelines for the interpretation of sequence variants: a joint consensus recommendation of the American College of Medical Genetics and Genomics and the Association for Molecular Pathology. Genet Med. 2015;17(5):405‐424. 25741868 10.1038/gim.2015.30 PMC4544753

[25] Riggs ER , Andersen EF, Cherry AM, et al Technical standards for the interpretation and reporting of constitutional copy-number variants: a joint consensus recommendation of the American College of Medical Genetics and Genomics (ACMG) and the Clinical Genome Resource (ClinGen). Genet Med. 2020;22(2):245‐257. 31690835 10.1038/s41436-019-0686-8 PMC7313390

[26] Karczewski KJ , Francioli LC, Tiao G, et al The mutational constraint spectrum quantified from variation in 141,456 humans. Nature. 2020;581(7809):434‐443. 32461654 10.1038/s41586-020-2308-7 PMC7334197

[27] Wright CF , FitzPatrick DR, Firth HV. Paediatric genomics: diagnosing rare disease in children. Nat Rev Genet. 2018;19(5):253‐268. 29398702 10.1038/nrg.2017.116

[28] Dentici ML , Di Pede A, Lepri FR, et al Kabuki syndrome: clinical and molecular diagnosis in the first year of life. Arch Dis Child. 2015;100(2):158‐164. 25281733 10.1136/archdischild-2013-305858

